# Bone-conducted ultrasonic auditory brainstem response thresholds in a mouse model of cisplatin-induced hearing loss

**DOI:** 10.1371/journal.pone.0353954

**Published:** 2026-07-16

**Authors:** Akihito Nakanishi, Noriko Nagase, Hirokazu Kousaki, Bakushi Ogawa, Kazuhiro Horii, Iori Niitsu Morimoto, Yuka Morita, Fumiaki Nin

**Affiliations:** 1 Division of Biological Principles, Department of Physiology and Biophysics, Graduate School of Medicine, Gifu University, 1-1 Yanagido, Gifu, Japan; 2 Department of Otolaryngology-Head and Neck Surgery, University of Toyama, Sugitani, Toyama, Japan; 3 Division of Sensorimotor Medicine, Department of Otolaryngology-Head and Neck Surgery, Graduate School of Medicine, Gifu University, 1-1 Yanagido, Gifu, Japan; Teikyo University Hospital Mizonokuchi, JAPAN

## Abstract

Cisplatin is widely used in cancer treatment but can cause hearing loss, predominantly at high frequencies. Auditory responses to bone-conducted ultrasonic stimulation have been proposed as a potential indicator of cochlear function, but it remains unclear how such responses are affected by cochlear damage. In this study, we measured auditory brainstem response (ABR) thresholds within and beyond the conventional hearing range in a mouse model of cisplatin-induced hearing loss. Of 32 mice initially examined, 9 were excluded at baseline because of pre-existing high-frequency hearing impairment. The remaining animals received either saline or cisplatin, and post-treatment analysis in the cisplatin group was based on 16 surviving mice. Cisplatin administration increased ABR thresholds within the conventional hearing range. In contrast, threshold shifts evoked by bone-conducted ultrasonic stimulation were often smaller. Paired comparison of mean threshold shifts showed that changes evoked by bone-conducted ultrasonic stimulation were significantly smaller than those within the conventional hearing range. These findings show that ABR threshold shifts evoked by bone-conducted ultrasonic stimulation do not necessarily parallel those within the conventional hearing range in cisplatin-treated mice.

## Introduction

Cisplatin was first synthesized in the 19th century and later recognized for its potent antitumor activity when platinum complexes were found to inhibit cell division [1]. These discoveries led to its clinical introduction in the 1970s, where it dramatically improved survival in several malignancies, including metastatic testicular cancer [2–4]. Today, cisplatin remains a cornerstone of treatment for solid tumors such as lung, gastric, and head and neck cancers [5,6]. However, its clinical utility is limited by dose-dependent toxicities, most notably nephrotoxicity and ototoxicity [7]. Cisplatin-induced hearing loss is typically bilateral, progressive, and irreversible, predominantly affecting high frequencies [8,9].

Current monitoring of cisplatin-induced hearing loss relies on serial audiometry or electrophysiology, which detect threshold shifts after onset [10,11]. This adverse effect is not only common—affecting up to 60% of patients in some regimens—but also permanent, significantly impairing communication, social interaction, and quality of life [8,10,11]. In pediatric and young adult populations, the consequences extend to language development, education, and long-term psychosocial outcomes [12,13]. However, there is still no effective method to restore hearing once it has been lost.

Ultrasonic hearing—perception evoked by bone-conducted ultrasonic stimulation beyond the conventional hearing range—offers a potential window into cochlear integrity [14,15]. Bone-conducted ultrasonic stimulation evokes auditory brainstem responses (ABR) and cochlear microphonics in rodents and humans, implicating canonical auditory pathways. Indeed, several studies have explored hearing-assistive approaches that utilize ultrasonic perception [16–20]. In addition, recent work suggests that this phenomenon depends on cochlear mechanisms, including responses of hair cells in the hook region to harmonic components of the sound that fall within the conventional hearing range [14,15]. This principle, termed harmonics detection, clarifies the basis of ultrasonic hearing under normal conditions. Although several studies have examined the mechanisms of ultrasonic hearing under normal conditions, the effects of cochlear damage on ABR evoked by bone-conducted ultrasonic stimulation have not been systematically investigated.

This gap is clinically relevant because cisplatin preferentially damages basal cochlear regions encoding high frequencies, which may also influence responses to bone-conducted ultrasonic stimulation. The present study was therefore designed as a functional and phenomenological investigation of how cochlear damage differentially affects ABR thresholds within the conventional hearing range and those evoked by bone-conducted ultrasonic stimulation.

Using a mouse model, we examined how ABR thresholds evoked by bone-conducted ultrasonic stimulation change relative to those within the conventional hearing range after cisplatin administration. Rather than directly identifying the underlying mechanism, we aimed to determine whether cochlear dysfunction produced by cisplatin differentially affects these responses and thereby constrains possible mechanisms of bone-conducted ultrasonic hearing.

## Materials and methods

### Ethical approval

The animals were treated in compliance with the Guiding Principles for the Care and Use of Animals in the field of Physiological Science set by the Physiological Society of Japan. The experimental protocol was approved by the Institutional Animal Research Committee of Gifu University (Approval No. 20220011). All animal handling and procedures adhered to the Animal Research: Reporting of in vivo Experiments (ARRIVE) guidelines. C57BL/6JmsSlc mice (6–12 weeks of age) from SLC Inc., Hamamatsu, Japan, were housed at the animal facility under a 12-hr light/12-hr dark cycle (lights on 7:00–19:00) with ad libitum access to laboratory chow and water. Animals were handled by personnel trained according to standard institutional procedures for animal care and experimentation.

### Animals and experimental procedures

The study included a total of 32 healthy female mice weighing 20–25 g. Female mice were used to maintain consistency with our previous studies using the same bone-conducted ultrasonic ABR paradigm in mice [15]. For ABR measurements, animals were anesthetized with an intraperitoneal injection of a mixture of medetomidine (0.3 mg/kg), midazolam (4.0 mg/kg), and butorphanol (5.0 mg/kg) [15]. Body temperature was monitored with a rectal thermometer and maintained with a heating pad. Supplemental doses of anesthesia were administered as needed to ensure the absence of a pedal withdrawal reflex to toe pinch. At the end of the experiment, the anesthetized animals were euthanized by decapitation. Nine animals with a hearing threshold of ≥70 dB at 40 kHz during the initial ABR measurement were excluded at baseline according to a predefined criterion to eliminate animals with pre-existing high-frequency hearing impairment. The remaining 23 animals entered the experiment, of which 5 received saline and 18 received cisplatin. ABR was measured before treatment and again on day 8, and cisplatin was administered intraperitoneally at 5 mg/kg for 3 consecutive days. The cisplatin regimen was selected based on previous mouse studies using repeated systemic cisplatin administration to induce hearing loss, as well as on the known dose-dependent nature of cisplatin ototoxicity [7,21]. Two animals died after cisplatin administration; therefore, post-treatment analysis in the cisplatin group was based on 16 animals. Animals were monitored at least daily throughout the experimental period and more frequently after cisplatin administration for general condition, body weight, posture, spontaneous activity, food and water intake, and signs of distress. Humane endpoints were predefined such that animals showing severe or persistent distress, marked reduction in activity, inability to access food or water, or substantial body weight loss would be euthanized immediately. No animals met these predefined humane endpoints before the scheduled endpoint. All efforts were made to minimize suffering throughout the study. Two animals died unexpectedly after cisplatin administration, and the precise cause of death was not determined. All surviving animals were euthanized under anesthesia by decapitation at the end of the experiment.

### Calibration of bone-conducted sound and ultrasound stimulation

We used a piezoelectric actuator (PA44LEW; Thorlabs, US) as a stimulator, which was driven by a function generator (Model WF1948; NF Corporation, Japan). For pressure calibration, we used a high-sensitivity hydrophone (Model TC4034, Teledyne Marine REASON, US) with an amplifier (Model EC6081 mk2, Teledyne Marine REASON, US). The basic aspects of bone-conducted ultrasonic stimulation were similar to those previously reported [14,15]. A compression force of 0.5 N was maintained on the actuator tip using a force gauge. The actuator tip was attached to the temporal bone. To maintain the correct angle of the stimulator on the temporal bone, we attached a cone-shaped tip to the ceramic rod (S1A, S1B Fig). The hydrophone was used to calibrate the actuator output under the same driving conditions as those used in the experiment, thereby providing a standardized reference for stimulus intensity. To enable direct comparison of auditory thresholds between air and bone conduction, all pressure levels were expressed in decibels with a standard bone compression pressure of 2 mPa, as previously reported [14,15]. This reference was used as a conventional reporting scale for bone-conducted stimulation rather than as a direct estimate of the mechanical load applied to the cochlea. In addition, because the stimulator tip was applied to the temporal bone, the stimulation could in principle include a minor airborne component; however, in our recordings the tympanic membrane was perforated as shown in S1B Fig in S1 Text, minimizing the contribution of air conduction.

### Electrophysiological measurements

The basic method for ABR recording was the same as previously described [14,15]. Stainless-steel needle electrodes were placed subcutaneously in the postauricular region and the neck. For the stimuli, 6-ms tone bursts including 0.5 ms linear rise and fall times at frequencies of 16, 40, 103, 145, 202, and 244 kHz were generated by the function generator. Among these frequencies, 16, 40, and 103 kHz were classified as within the conventional hearing range, whereas 145, 202, and 244 kHz were classified as frequencies beyond the conventional hearing range and were presented by bone conduction. Acquired data were recorded by a digitizer with a sampling frequency of 2.4 MHz (Model PCIe-6374; National Instruments, US). Five hundred sweeps were averaged using a custom-made LabVIEW program (LabVIEW 2019 SP2; National Instruments, US). Individual signals were amplified 5,000-fold and processed with an analog bandpass filter (300−1,000 Hz) in a commercial amplifier (Model 3000, A-M Systems, US). ABR thresholds were defined as the lowest pressure level at which wave III was reliably detected because this component was the most consistently identifiable across the tested frequencies under our recording conditions [15]. Wave III was identified by visual inspection of the averaged ABR waveforms as a repeatable positive-negative deflection occurring at the expected latency range under identical recording conditions, and the same criterion was applied across animals and stimulus frequencies.

### Statistical analysis

**Fig 1:**
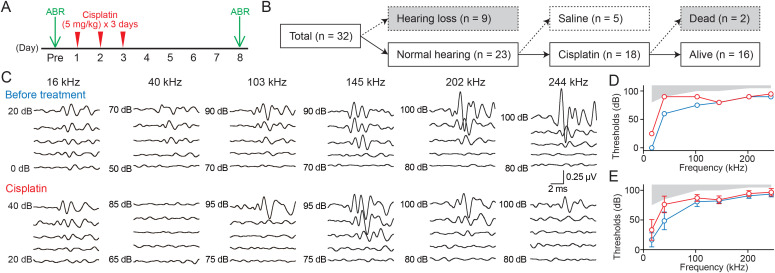
Experimental design and changes in ABR thresholds after cisplatin administration. (A) Experimental timeline for ABR measurements and cisplatin administration. (B) Flow diagram of animal allocation and analysis. (C) Representative ABR waveforms across frequencies within and beyond the conventional hearing range before and after cisplatin administration. For each stimulus frequency, upper traces show recordings obtained before treatment and lower traces show recordings obtained after cisplatin treatment. Waveforms are displayed in 5 dB decrements. (D) Threshold profile across frequencies in the representative animal shown in (C). (E) Group data of ABR thresholds across frequencies before and after cisplatin administration in the analyzed mice (n = 16). Blue and red lines indicate thresholds before and after cisplatin administration, respectively. Circles and error bars represent mean values and standard deviations. The gray shaded area indicates stimulus levels that could not be generated by the stimulation system.

**Fig 2:**
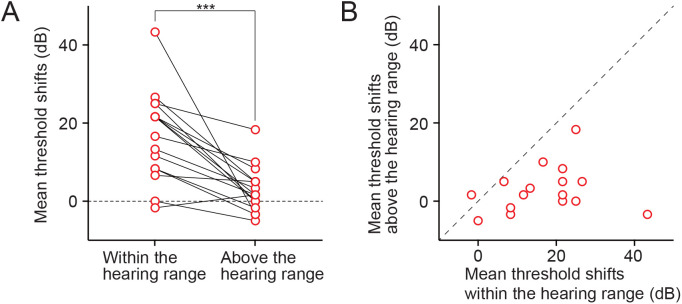
Relationship between threshold shifts within and beyond the conventional hearing range after cisplatin administration. (A) Paired comparison of mean threshold shifts within and beyond the conventional hearing range in individual cisplatin-treated mice (n = 16). Threshold shifts were calculated as post-treatment minus pre-treatment values and averaged across frequencies within each range. Threshold shifts beyond the conventional hearing range were significantly smaller than those within the conventional hearing range (paired t-test, p = 0.0002). (B) Scatter plot of mean threshold shifts within versus beyond the conventional hearing range in individual cisplatin-treated mice. Each point represents one animal. The dashed line indicates equality between threshold shifts within and beyond the conventional hearing range.

Threshold differences were calculated as post-treatment minus pre-treatment values. For Fig 1E, threshold differences were averaged across all analyzed animals at each frequency, and descriptive statistics are presented as means ± SD. For Fig 2, threshold differences were averaged across frequencies within each animal for the conventional hearing range (16, 40, and 103 kHz) and for frequencies beyond the conventional hearing range (145, 202, and 244 kHz). Mean threshold shifts within and beyond the conventional hearing range were compared using paired t-test. Sample sizes (n) are indicated in the main text and figure legends. A two-sided p value of <0.05 was considered statistically significant. All statistical analyses were performed using Prism 10 (GraphPad Software, USA).

## Results

### Cisplatin administration protocol and ABR measurements within and beyond the conventional hearing range

Bone-conducted ultrasonic stimulation at frequencies beyond the conventional hearing range evoked ABR in mice [15]. The experimental design is summarized in Fig 1A. Of 32 mice initially examined, 9 were excluded at baseline because of pre-existing high-frequency hearing impairment, and the remaining 23 animals entered the experiment. Among these, 5 received saline and 18 received cisplatin, and post-treatment analysis in the cisplatin group was based on 16 surviving animals (Fig 1B). In saline-treated mice, representative waveforms and frequency-specific thresholds showed no significant changes across all tested frequencies (S2A, S2B Fig in S1 Text). This finding was consistent across five animals (S2C Fig in S1 Text). Representative ABR waveforms recorded before and after cisplatin administration are shown in Fig 1C. In the representative animal, threshold elevations were observed at 16, 40, and 103 kHz after cisplatin treatment, while little or no elevation was observed at 145 kHz, 202 kHz, and 244 kHz during bone-conducted ultrasonic stimulation (Fig 1D). Consistent with previous reports [8], threshold elevations were more pronounced at 40 kHz than at 16 kHz, except at 103 kHz near the upper limit of the conventional hearing range. Group data from the analyzed cisplatin-treated mice are shown in Fig 1E (n = 16).

### Relationship between threshold shifts within and beyond the conventional hearing range after cisplatin administration

To compare the effects of cisplatin across frequency ranges, threshold shifts were averaged within the conventional hearing range and at frequencies beyond the conventional hearing range for each animal. Paired comparison of these mean values showed that threshold shifts at frequencies beyond the conventional hearing range were generally smaller than those within the conventional hearing range in individual cisplatin-treated mice (Fig 2A). This relationship is further illustrated in the scatter plot in Fig 2B, in which most animals were distributed below the line of equality, indicating smaller threshold shifts at frequencies beyond the conventional hearing range than within the conventional hearing range. Together, these findings show that threshold elevations at frequencies beyond the conventional hearing range did not necessarily parallel those observed within the conventional hearing range after cisplatin administration.

## Discussion

In this study, we measured ABR using both stimuli within the conventional hearing range and bone-conducted ultrasonic stimulation and found that, even when hearing thresholds within the conventional hearing range were elevated after cisplatin administration, thresholds evoked by bone-conducted ultrasonic stimulation beyond the conventional hearing range sometimes showed little or no change. These findings indicate that auditory responses evoked by bone-conducted ultrasonic stimulation beyond the conventional hearing range do not necessarily parallel threshold elevations within the conventional hearing range in cisplatin-treated mice. Although ultrasonic hearing is thought to depend on harmonics detection by hair cells responding to components within the conventional hearing range [14,15], the present findings suggest that cochlear dysfunction does not affect these two response domains in a strictly parallel manner, thereby placing constraints on possible mechanisms of bone-conducted ultrasonic hearing without directly establishing one.

This dissociation may be relevant when evaluating cochlear function across different stimulus conditions, because responses evoked by bone-conducted ultrasonic stimulation may not simply reflect the degree of impairment observed within the conventional hearing range. At the same time, the present study was designed to describe this physiological observation rather than to determine its underlying mechanism. Although the sample size was limited, the preservation of responses evoked by bone-conducted ultrasonic stimulation in the majority of affected animals suggests that the observation is unlikely to be explained solely by random variability. Further studies will be required to clarify the basis of this finding.

An important limitation of the present study is that we did not systematically examine the dose–response relationship of cisplatin ototoxicity. Cisplatin-induced hearing loss is known to be dose-dependent, and both the severity and time course of auditory dysfunction may vary according to cumulative dose and dosing schedule [7,8]. In addition, the baseline exclusion rate due to pre-existing high-frequency hearing impairment was relatively high. The cause of this pre-existing impairment was not determined in the present study. To reduce this source of variability, we applied a predefined exclusion criterion and limited the analysis to animals that met the baseline hearing requirement. Accordingly, the extent to which the preservation of responses evoked by bone-conducted ultrasonic stimulation beyond the conventional hearing range observed in this study generalizes across different cisplatin regimens or broader baseline populations remains unclear. Further studies should determine whether this dissociation is preserved across different dosing conditions and levels of cochlear dysfunction.

### Use of AI-assisted technologies

During the preparation of this manuscript, the authors used OpenAI ChatGPT (GPT-5.2, OpenAI, San Francisco, CA, USA; used in 2026) solely for assistance with English grammar, clarity, and stylistic refinement of the text. The AI tool did not generate or modify any scientific content, data, analyses, or interpretations. The authors take full responsibility for the integrity and accuracy of the manuscript.

## Supporting information

S1 Text Supplementary figures showing the bone-conducted ultrasound stimulation system and ABR thresholds in saline-treated control mice. (DOCX)
