# Correction: Bone-conducted ultrasonic auditory brainstem response thresholds in a mouse model of cisplatin-induced hearing loss

**DOI:** 10.1371/journal.pone.0358064

**Published:** 2026-09-09

**Authors:** Akihito Nakanishi, Noriko Nagase, Hirokazu Kousaki, Bakushi Ogawa, Kazuhiro Horii, Iori Niitsu Morimoto, Yuka Morita, Fumiaki Nin

The hyperlink in the Data Availability statement is incorrect. The correct hyperlink is: https://doi.org/10.6084/m9.figshare.31320841.
