# Supplementary material for: Bone-conducted ultrasonic auditory brainstem response thresholds in a mouse model of cisplatin-induced hearing loss
Source: PLoS One. 2026 Jul 16;21(7):e0353954. doi: 10.1371/journal.pone.0353954 (PMC13374921; doi:10.1371/journal.pone.0353954)
Supplement: S1 Text — (DOCX) [file pone.0353954.s001.docx]

**SI Text for**

Bone-conducted ultrasonic auditory brainstem response thresholds in a mouse model of cisplatin-induced hearing loss

Akihito Nakanishi, Noriko Nagase, Hirokazu Kousaki, Bakushi Ogawa, Kazuhiro Horii, Iori Niitsu Morimoto, Yuka Morita, Fumiaki Nin.

**This PDF includes:**

Figures S1 to S2

**
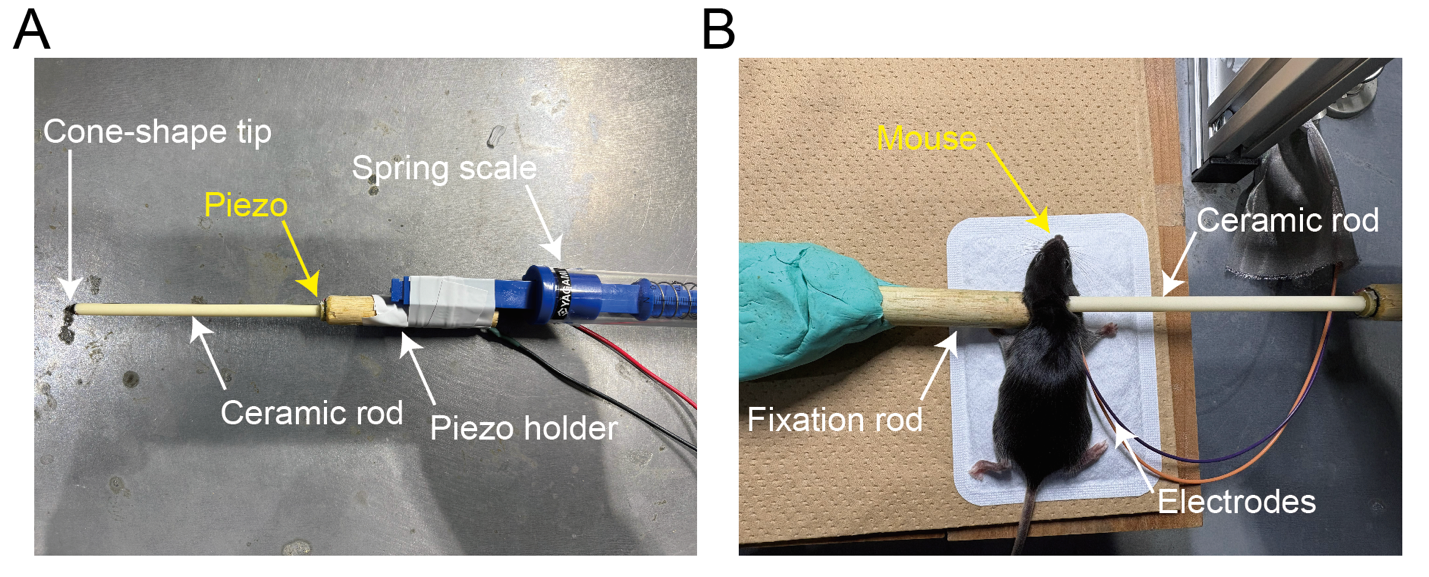
**

**Figure S1. Bone-conducted ultrasound stimulation system and experimental setup.** (A) Photograph of the piezoelectric stimulation assembly. The actuator was connected to a ceramic rod with a cone-shaped tip, and a spring scale was used to maintain a constant compression force. (B) Experimental setup during ABR recording. The ceramic rod was positioned against the temporal bone of the anesthetized mouse, and the cone-shaped tip was used to maintain stable contact at the stimulation site. The tympanic membrane was perforated to eliminate airborne sound components, as described previously (15). Two electrodes were placed subcutaneously for ABR measurements.

**
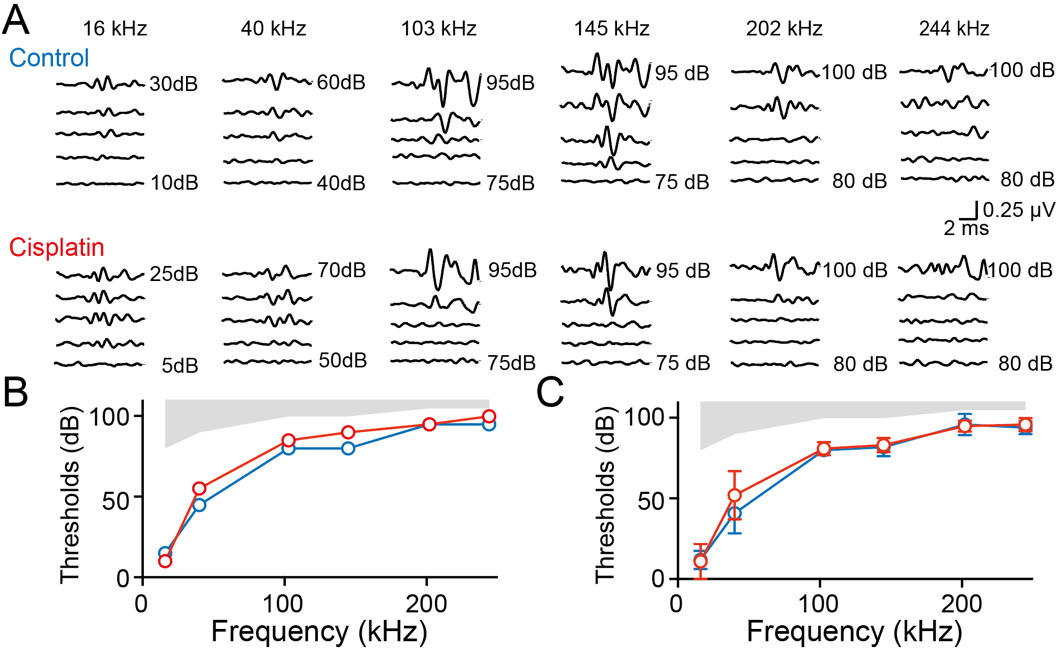
**

**Figure S2. ABR thresholds before and after saline administration in control mice.** (A) Representative ABR waveforms across frequencies within and beyond the conventional hearing range before and after saline administration. For each stimulus frequency, upper traces show control recordings and lower traces show recordings obtained 7 days after three consecutive daily intraperitoneal saline injections. Waveforms are displayed in 5 dB decrements. (B) Threshold profile across frequencies in the representative animal shown in (A). (C) Group data of ABR thresholds across frequencies in control mice before and after saline administration (n = 5). Lines indicate mean values, and error bars indicate standard deviations. The gray shaded area indicates stimulus levels that could not be generated by the stimulation system.
