# Supplementary material for: Bone-conducted ultrasonic auditory brainstem response thresholds in a mouse model of cisplatin-induced hearing loss
Source: PLoS One. 2026 Jul 16;21(7):e0353954. doi: 10.1371/journal.pone.0353954 (PMC13374921; doi:10.1371/journal.pone.0353954)
Supplement: Attachment — Submitted filename: Response_to_Reviewers_PONE-D-26-20660.docx [file pone.0353954.s003.docx]

**Response to Reviewers**

**Manuscript title:** Bone-conducted ultrasonic auditory brainstem response thresholds in a mouse model of cisplatin-induced hearing loss

**Manuscript number:** PONE-D-26-20660

We sincerely thank the Editor and the reviewers for their careful evaluation of our manuscript and for their constructive comments. We have revised the manuscript accordingly and believe that these changes have improved its clarity and overall presentation. Below, we respond point by point to each comment. All changes are reflected in the revised manuscript and highlighted in the tracked-changes version.

**Comment:** Please remove funding-related text from the manuscript, update the Funding Statement, and provide a Role of Funder statement.

**Response:** We have removed funding-related text from the manuscript. The funding information has been provided for updating the Funding Statement in the submission system. We have also provided the following Role of Funder statement in the accompanying cover letter: “The funders had no role in study design, data collection and analysis, decision to publish, or preparation of the manuscript.”

**Comment:** Please provide a direct link in the Data Availability Statement.

**Response:** We have revised the Data Availability Statement to include the direct repository link: “All data supporting the findings in this study are available within the paper, Supplementary Information, and figshare: https://doi.org/10.6084/m9.figshare.31429337”.

**Comment:** Please review any reviewer-suggested citations and ensure the reference list is correct and complete.

**Response:** We carefully reviewed the citations suggested or implied by the reviewers and checked the full reference list for completeness and accuracy.

**Response to Reviewer #1**

**Comment:** General assessment: The manuscript would benefit from improved clarity in its conceptual framework and interpretation. I recommend minor revision.

**Response:** We thank the reviewer for the positive assessment of the technical soundness of the study and for the helpful suggestions. We have revised the Introduction and Discussion to clarify the primary objective and positioning of the study, and we have strengthened the interpretation while keeping the scope appropriately functional and phenomenological.

**Comment:** 1. Clarification of the study objective and positioning. Please explicitly state the primary aim of the study and align the Introduction and Discussion accordingly.

**Response:** We agree and have revised both the Introduction and the Discussion to clarify the primary aim of the study. The revised Introduction now explicitly states that the study was designed as a functional and phenomenological investigation of how cochlear damage differentially affects auditory brainstem response thresholds within the conventional hearing range and those evoked by bone-conducted ultrasonic stimulation. We also clarified that the purpose was not to directly identify the underlying mechanism, but rather to determine whether cochlear dysfunction differentially affects these responses and thereby constrains possible mechanisms of bone-conducted ultrasonic hearing. The opening paragraph of the Discussion was revised accordingly to align the interpretation with this framing.

**Comment:** 2. Strengthening the Discussion by integrating existing knowledge, including cisplatin pathophysiology and current hypotheses regarding bone-conducted ultrasonic hearing.

**Response:** We have revised the Discussion to better integrate the present findings with existing knowledge. In particular, we now more clearly relate the results to the known dose-dependent nature and frequency dependence of cisplatin-induced cochlear dysfunction, as well as to current ideas regarding harmonics detection and bone-conducted ultrasonic hearing. We also clarified that the dissociation observed in the present study places constraints on possible mechanisms without directly establishing one.

**Comment:** Minor comment 1. Some parts of the Results section repeat methodological details. These descriptions should be consolidated in the Methods section. Please also add references to the corresponding figures/tables at appropriate locations in the text.

**Response:** We agree and have streamlined the Results section. Details regarding the cisplatin regimen, timing of measurements, and justification for the regimen were consolidated in Materials and Methods. The Results section now focuses more directly on the findings, and figure references were checked and retained at the relevant locations.

**Comment:** Minor comment 2. In Figure 1C, the label “control” appears to refer to recordings obtained before cisplatin administration rather than a saline-treated control group. Please revise the labeling.

**Response:** We agree and have revised the wording in Figure 1C and its legend. The previous wording “control” was replaced with “before treatment,” and the corresponding paired traces are now described as recordings obtained before treatment and after cisplatin treatment.

**Comment:** Minor comment 3. Defining abbreviations such as conventional hearing range and bone-conducted ultrasonic stimulation would improve readability.

**Response:** We appreciate this suggestion. After careful consideration, we chose not to introduce additional abbreviations in the revised manuscript, because the number of core technical terms is limited and we felt that repeatedly spelling them out would maintain clarity for a broad readership. However, we revised the surrounding text to improve readability and reduce redundancy.

**Response to Reviewer #2**

**Comment:** General assessment: The study is well conceived and potentially impactful, but several methodological ambiguities and structural redundancies should be addressed.

**Response:** We thank the reviewer for the positive evaluation and constructive suggestions. We have revised the manuscript to clarify the calibration procedure, reduce overlap between Methods and Results, define the criteria used for wave III identification more explicitly, and discuss the relatively high baseline exclusion rate.

**Comment:** The method used to calibrate the bone-conducted ultrasonic stimulation is not sufficiently clear. Please explain how calibration was achieved using a hydrophone and clarify the rationale for using dB re 2 mPa in bone conduction.

**Response:** We agree and have expanded the calibration description in Materials and Methods. The revised text now explains that the hydrophone was used to calibrate the actuator output under the same driving conditions as those used in the experiment, thereby providing a standardized reference for stimulus intensity. We also clarified that the standard bone compression pressure of 2 mPa was used as a conventional reporting scale for bone-conducted stimulation rather than as a direct estimate of the mechanical load applied to the cochlea. In addition, we explicitly note that, although a minor airborne component could in principle contribute to stimulation, perforation of the tympanic membrane in our recordings minimized the contribution of air conduction.

**Comment:** There is noticeable redundancy between the Materials and Methods and Results sections, particularly regarding the descriptions of the experimental design and the cisplatin administration protocol.

**Response:** We agree and have revised the manuscript accordingly. Details of the experimental schedule, cisplatin dosing regimen, and the rationale for the regimen were consolidated in Materials and Methods, and the Results section was shortened so that it focuses more directly on the experimental findings.

**Comment:** Regarding the electrophysiological measurements, what specific criteria were used to determine the presence or absence of auditory brainstem response wave III? Please also provide information regarding intra-individual reproducibility and consistency.

**Response:** We have clarified this point in the Materials and Methods. The revised text now states that wave III was identified by visual inspection of the averaged auditory brainstem response waveforms as a repeatable positive-negative deflection occurring at the expected latency range under identical recording conditions, and that the same criterion was applied across animals and stimulus frequencies. Because thresholds were assigned from averaged responses recorded under identical conditions, this same standardized criterion was used consistently throughout the study.

**Comment:** The exclusion rate of mice at baseline due to pre-existing high-frequency hearing impairment appears relatively high. Please discuss possible causes, including whether environmental noise might have influenced baseline thresholds.

**Response:** We agree that this point warrants discussion. We have added a statement to the Discussion noting that the baseline exclusion rate due to pre-existing high-frequency hearing impairment was relatively high, that the cause of this pre-existing impairment was not determined in the present study, and that a predefined exclusion criterion was used to reduce this source of variability and to limit the analysis to animals meeting the baseline hearing requirement.

We again thank the Editor and the reviewers for their thoughtful and constructive comments. We hope that the revisions satisfactorily address all concerns and that the revised manuscript will now be suitable for publication in PLOS ONE.
