# Supplementary material for: Bone-conducted ultrasonic auditory brainstem response thresholds in a mouse model of cisplatin-induced hearing loss
Source: PLoS One. 2026 Jul 16;21(7):e0353954. doi: 10.1371/journal.pone.0353954 (PMC13374921; doi:10.1371/journal.pone.0353954)
Supplement: Peer review history

# Peer review: Decision Letter 0

**DOI:** 10.1371/journal.pone.0353954.r001

**Authors:** Toru Miwa

11 Jun 2026

PONE-D-26-20660

Bone-conducted ultrasonic auditory brainstem response thresholds in a mouse model of cisplatin-induced hearing loss

PLOS One

Dear Dr. Nin,

Thank you for submitting your manuscript to PLOS ONE. After careful consideration, we feel that it has merit but does not fully meet PLOS ONE’s publication criteria as it currently stands. Therefore, we invite you to submit a revised version of the manuscript that addresses the points raised during the review process.

Please submit your revised manuscript by Jul 11 2026 11:59PM. If you will need more time than this to complete your revisions, please reply to this message or contact the journal office at plosone@plos.org. When you're ready to submit your revision, log on to https://www.editorialmanager.com/pone/ and select the 'Submissions Needing Revision' folder to locate your manuscript file.

Please include the following items when submitting your revised manuscript:

- A letter that responds to each point raised by the academic editor and reviewer(s). You should upload this letter as a separate file labeled 'Response to Reviewers'.
- A marked-up copy of your manuscript that highlights changes made to the original version. You should upload this as a separate file labeled 'Revised Manuscript with Track Changes'.
- An unmarked version of your revised paper without tracked changes. You should upload this as a separate file labeled 'Manuscript'.

If you would like to make changes to your financial disclosure, please include your updated statement in your cover letter. Guidelines for resubmitting your figure files are available below the reviewer comments at the end of this letter.

If applicable, we recommend that you deposit your laboratory protocols in protocols.io to enhance the reproducibility of your results. Protocols.io assigns your protocol its own identifier (DOI) so that it can be cited independently in the future. For instructions see: https://journals.plos.org/plosone/s/submission-guidelines#loc-laboratory-protocols. Additionally, PLOS ONE offers an option for publishing peer-reviewed Lab Protocol articles, which describe protocols hosted on protocols.io. Read more information on sharing protocols at https://plos.org/protocols?utm_medium=editorial-email&utm_source=authorletters&utm_campaign=protocols.

As the corresponding author, your ORCID iD is verified in the submission system and will appear in the published article. PLOS supports the use of ORCID, and we encourage all coauthors to register for an ORCID iD and use it as well. Please encourage your coauthors to verify their ORCID iD within the submission system before final acceptance, as unverified ORCID iDs will not appear in the published article. *Only*  the individual author can complete the verification step; PLOS staff *cannot*  verify ORCID iDs on behalf of authors.

We look forward to receiving your revised manuscript.

Kind regards,

Toru Miwa

Academic Editor

PLOS One

Journal Requirements:

When submitting your revision, we need you to address these additional requirements.

1. Please ensure that your manuscript meets PLOS ONE's style requirements, including those for file naming. The PLOS ONE style templates can be found at

https://journals.plos.org/plosone/s/file?id=wjVg/PLOSOne_formatting_sample_main_body.pdf and

https://journals.plos.org/plosone/s/file?id=ba62/PLOSOne_formatting_sample_title_authors_affiliations.pdf

2. Thank you for stating the following in the Acknowledgments Section of your manuscript:

“This study was supported by the Suzuken Memorial Foundation 23-022 (to F.N.), the Naito Foundation (to F.N.), Terumo Life Science Foundation 24-III1047 (to F.N.), the Mitsubishi Foundation 202510041 (to F.N.), Takeda Science Foundation 2024047242 (to K.H.), Ogawa Memorial Foundation (to K.H.), the Japan Agency for Medical Research and Development (AMED) A-281 (to F.N.), JST FOREST Program 25126424 (to F.N.), and JSPS KAKENHI JP24K02596 (to F.N.) and JP25K20163 (to B.O.).”

We note that you have provided additional information within the Acknowledgements Section that is not currently declared in your Funding Statement. Please note that funding information should not appear in the Acknowledgments section or other areas of your manuscript. We will only publish funding information present in the Funding Statement section of the online submission form.

Please remove any funding-related text from the manuscript and let us know how you would like to update your Funding Statement. Currently, your Funding Statement reads as follows:

“This study was supported by the Suzuken Memorial Foundation 23-022 (to F.N.), the Naito Foundation (to F.N.), Terumo Life Science Foundation 24-III1047 (to F.N.), the Mitsubishi Foundation 202510041 (to F.N.), Takeda Science Foundation 2024047242 (to K.H.), Ogawa Memorial Foundation (to K.H.), the Japan Agency for Medical Research and Development (AMED) A-281 (to F.N.), JST FOREST Program 25126424 (to F.N.), and JSPS KAKENHI JP24K02596 (to F.N.) and JP25K20163 (to B.O.).”

Please include your amended statements within your cover letter; we will change the online submission form on your behalf.

3. Thank you for stating the following financial disclosure:

“This study was supported by the Suzuken Memorial Foundation 23-022 (to F.N.), the Naito Foundation (to F.N.), Terumo Life Science Foundation 24-III1047 (to F.N.), the Mitsubishi Foundation 202510041 (to F.N.), Takeda Science Foundation 2024047242 (to K.H.), Ogawa Memorial Foundation (to K.H.), the Japan Agency for Medical Research and Development (AMED) A-281 (to F.N.), JST FOREST Program 25126424 (to F.N.), and JSPS KAKENHI JP24K02596 (to F.N.) and JP25K20163 (to B.O.).”

Please state what role the funders took in the study.  If the funders had no role, please state: "The funders had no role in study design, data collection and analysis, decision to publish, or preparation of the manuscript."

If this statement is not correct you must amend it as needed.

Please include this amended Role of Funder statement in your cover letter; we will change the online submission form on your behalf.

4. Please note that your Data Availability Statement is currently missing a direct link to access each database. If your manuscript is accepted for publication, you will be asked to provide these details on a very short timeline. We therefore suggest that you provide this information now, though we will not hold up the peer review process if you are unable.

5. If the reviewer comments include a recommendation to cite specific previously published works, please review and evaluate these publications to determine whether they are relevant and should be cited. There is no requirement to cite these works unless the editor has indicated otherwise.

Please review your reference list to ensure that it is complete and correct. If you have cited papers that have been retracted, please include the rationale for doing so in the manuscript text, or remove these references and replace them with relevant current references. Any changes to the reference list should be mentioned in the rebuttal letter that accompanies your revised manuscript. If you need to cite a retracted article, indicate the article’s retracted status in the References list and also include a citation and full reference for the retraction notice.

[Note: HTML markup is below. Please do not edit.]

Reviewers' comments:

Reviewer's Responses to Questions

**Comments to the Author**

1. Is the manuscript technically sound, and do the data support the conclusions?

The manuscript must describe a technically sound piece of scientific research with data that supports the conclusions. Experiments must have been conducted rigorously, with appropriate controls, replication, and sample sizes. The conclusions must be drawn appropriately based on the data presented.

Reviewer #1: Yes

Reviewer #2: Yes

**********

2. Has the statistical analysis been performed appropriately and rigorously?

Reviewer #1: Yes

Reviewer #2: Yes

**********

3. Have the authors made all data underlying the findings in their manuscript fully available?

The PLOS Data policy requires authors to make all data underlying the findings described in their manuscript fully available without restriction, with rare exception (please refer to the Data Availability Statement in the manuscript PDF file). The data should be provided as part of the manuscript or its supporting information, or deposited to a public repository. For example, in addition to summary statistics, the data points behind means, medians and variance measures should be available. If there are restrictions on publicly sharing data—e.g. participant privacy or use of data from a third party—those must be specified.

Reviewer #1: Yes

Reviewer #2: Yes

**********

4. Is the manuscript presented in an intelligible fashion and written in standard English?

PLOS ONE does not copyedit accepted manuscripts, so the language in submitted articles must be clear, correct, and unambiguous. Any typographical or grammatical errors should be corrected at revision, so please note any specific errors here.

Reviewer #1: Yes

Reviewer #2: Yes

**********

5. Review Comments to the Author

Please use the space provided to explain your answers to the questions above. You may also include additional comments for the author, including concerns about dual publication, research ethics, or publication ethics. (Please upload your review as an attachment if it exceeds 20,000 characters)

Reviewer #1: ・General assessment

This manuscript investigates auditory brainstem responses (ABRs) evoked by bone-conducted ultrasonic stimulation in a mouse model of cisplatin-induced hearing loss. The finding that ultrasonic-evoked ABR thresholds are relatively preserved compared to those within the conventional hearing range is intriguing and potentially important. It suggests that ultrasonic-evoked responses may not simply reflect cochlear function in a tonotopic manner.

Although I do not consider additional experiments to be necessary and the data appear technically sound, the manuscript would benefit from improved clarity in its conceptual framework and interpretation. These issues can be addressed through revision of the text. Therefore, I recommend minor revision.

・Major comments

1. Clarification of the study objective and positioning

The overall aim and positioning of the present study require further clarification.

As currently written, the manuscript can be interpreted in multiple ways:

(1) as a comparative study between conventional and ultrasonic ABR,

(2) as a clinically oriented attempt to explore alternative indicators for cisplatin-induced hearing loss, or

(3) as an investigation into the mechanisms of bone-conducted ultrasonic hearing.

While each of these interpretations is potentially valid, the manuscript does not clearly define which represents the primary objective. This ambiguity makes it difficult to fully appreciate the significance of the findings.

In particular, if the study is framed merely as a comparison between stimulus modalities, the rationale for employing a cisplatin-induced hearing loss model becomes less clear. Instead, the present design appears more suitable for examining how cochlear damage differentially affects these responses, thereby providing constraints on possible mechanisms.

Given the absence of morphological or cellular analyses, the study is best positioned as a functional and phenomenological investigation that constrains possible mechanisms rather than directly elucidating them.

I therefore encourage the authors to explicitly state the primary aim of the study and align the Introduction and Discussion accordingly.

2. Strengthening the Discussion by integrating existing knowledge

The authors state that the purpose of this study is to describe physiological findings rather than to elucidate mechanisms. While this is a reasonable scope, the Discussion would benefit from a more structured and integrative interpretation of the results.

In particular, it would be helpful to relate the present findings to:

(i) the known pathophysiology of cisplatin-induced cochlear damage (e.g., preferential basal turn involvement and outer hair cell vulnerability), and

(ii) current hypotheses regarding bone-conducted ultrasonic hearing (e.g., harmonic distortion vs alternative cochlear or mechanical mechanisms).

The observed dissociation between conventional ABR and ultrasonic-evoked ABR appears difficult to reconcile with the known tonotopic vulnerability of the cochlea. This point is highly interesting but not sufficiently explored.

Without requiring additional experiments, discussing how the present results align with or challenge these frameworks would clarify the significance of the findings and strengthen the manuscript.

・Minor comments

1. Some parts of the Results section repeat methodological details. These descriptions should be consolidated in the Methods section. Please also add references to the corresponding figures/tables at appropriate locations in the text.

2. In Figure 1C, the label “control” appears to refer to recordings obtained before cisplatin administration rather than a saline-treated control group. To avoid confusion, please revise the labeling (e.g., “before treatment”) and clearly distinguish it from saline controls.

3. The terms “conventional hearing range” and “bone-conducted ultrasonic stimulation (beyond the conventional hearing range)” are repeatedly used and relatively long. Defining appropriate abbreviations (e.g., CHR and BCUS) would improve readability.

Reviewer #2: This manuscript investigates the threshold shifts of auditory brainstem responses (ABRs) to stimuli within the conventional hearing range and to bone-conducted ultrasonic stimulation using a mouse model of cisplatin-induced hearing loss. The authors demonstrate that while conventional hearing thresholds significantly increased after cisplatin administration, the threshold elevations evoked by bone-conducted ultrasonic stimulation remained relatively small. This dissociation between conventional and ultrasonic hearing under cochlear dysfunction is highly intriguing and opens a novel perspective in auditory research. Overall, the study is well-conceived and potentially impactful. However, there are several methodological ambiguities and structural redundancies that need to be addressed before publication.

Specific Comments:

The method used to calibrate the bone-conducted ultrasonic stimulation is not sufficiently clear. Although the authors cite previous literature, the specific procedure in the context of this study remains ambiguous. Because the output force of a piezoelectric actuator is heavily dependent on the mechanical impedance of the object it is attached to, it is critical to explain how the calibration was accurately achieved using a hydrophone. Furthermore, since the stimulus intensity is expressed as an absolute value in decibels relative to a standard bone compression pressure of 2 mPa (dB re 2 mPa), the theoretical rationale and specific determination method for using this pressure-based reference in bone conduction should be explicitly detailed in the text.The method used to calibrate the bone-conducted ultrasonic stimulation is not sufficiently clear. Although the authors cite previous literature, the specific procedure in the context of this study remains ambiguous. Because the output force of a piezoelectric actuator is heavily dependent on the mechanical impedance of the object it is attached to, it is critical to explain how the calibration was accurately achieved using a hydrophone. Furthermore, since the stimulus intensity is expressed as an absolute value in decibels relative to a standard bone compression pressure of 2 mPa (dB re 2 mPa), the theoretical rationale and specific determination method for using this pressure-based reference in bone conduction should be explicitly detailed in the text.

There is noticeable redundancy between the "Materials and Methods" and "Results" sections, particularly regarding the descriptions of the experimental design and the cisplatin administration protocol (e.g., dosage and consecutive days). To improve readability and flow, the authors should streamline these sections and ensure that the "Results" section focuses primarily on findings rather than repeating the experimental procedures.

Regarding the electrophysiological measurements, what specific criteria (e.g., visual inspection guidelines or signal-to-noise ratio thresholds) were used to reliably determine the presence or absence of ABR wave III? In addition, please provide information or data regarding the intra-individual reproducibility and consistency of these wave measurements across trials.

The exclusion rate of mice at baseline due to pre-existing high-frequency hearing impairment appears relatively high (9 out of 32 mice, ~28%). Could the authors discuss the potential causes of this pre-existing impairment? Specifically, is there a possibility that environmental noise within the animal housing facility influenced the baseline hearing thresholds of these young mice?

**********

6. PLOS authors have the option to publish the peer review history of their article (what does this mean?). If published, this will include your full peer review and any attached files.

If you choose “no”, your identity will remain anonymous but your review may still be made public.

**Do you want your identity to be public for this peer review?**  For information about this choice, including consent withdrawal, please see our Privacy Policy.

Reviewer #1: No

Reviewer #2: **Yes:**  Takuji Koike

**********

[NOTE: If reviewer comments were submitted as an attachment file, they will be attached to this email and accessible via the submission site. Please log into your account, locate the manuscript record, and check for the action link "View Attachments". If this link does not appear, there are no attachment files.]

To ensure your figures meet our technical requirements, please review our figure guidelines: https://journals.plos.org/plosone/s/figures

You may also use PLOS’s free figure tool, NAAS, to help you prepare publication quality figures: https://journals.plos.org/plosone/s/figures#loc-tools-for-figure-preparation.

NAAS will assess whether your figures meet our technical requirements by comparing each figure against our figure specifications.

# Author comment: Author response to Decision Letter 1

**DOI:** 10.1371/journal.pone.0353954.r002

11 Jun 2026

Comment: Please remove funding-related text from the manuscript, update the Funding Statement, and provide a Role of Funder statement.

Response: We have removed funding-related text from the manuscript. The funding information has been provided for updating the Funding Statement in the submission system. We have also provided the following Role of Funder statement in the accompanying cover letter: “The funders had no role in study design, data collection and analysis, decision to publish, or preparation of the manuscript.”

Comment: Please provide a direct link in the Data Availability Statement.

Response: We have revised the Data Availability Statement to include the direct repository link: “All data supporting the findings in this study are available within the paper, Supplementary Information, and figshare: https://doi.org/10.6084/m9.figshare.31429337”.

Comment: Please review any reviewer-suggested citations and ensure the reference list is correct and complete.

Response: We carefully reviewed the citations suggested or implied by the reviewers and checked the full reference list for completeness and accuracy.

Response to Reviewer #1

Comment: General assessment: The manuscript would benefit from improved clarity in its conceptual framework and interpretation. I recommend minor revision.

Response: We thank the reviewer for the positive assessment of the technical soundness of the study and for the helpful suggestions. We have revised the Introduction and Discussion to clarify the primary objective and positioning of the study, and we have strengthened the interpretation while keeping the scope appropriately functional and phenomenological.

Comment: 1. Clarification of the study objective and positioning. Please explicitly state the primary aim of the study and align the Introduction and Discussion accordingly.

Response: We agree and have revised both the Introduction and the Discussion to clarify the primary aim of the study. The revised Introduction now explicitly states that the study was designed as a functional and phenomenological investigation of how cochlear damage differentially affects auditory brainstem response thresholds within the conventional hearing range and those evoked by bone-conducted ultrasonic stimulation. We also clarified that the purpose was not to directly identify the underlying mechanism, but rather to determine whether cochlear dysfunction differentially affects these responses and thereby constrains possible mechanisms of bone-conducted ultrasonic hearing. The opening paragraph of the Discussion was revised accordingly to align the interpretation with this framing.

Comment: 2. Strengthening the Discussion by integrating existing knowledge, including cisplatin pathophysiology and current hypotheses regarding bone-conducted ultrasonic hearing.

Response: We have revised the Discussion to better integrate the present findings with existing knowledge. In particular, we now more clearly relate the results to the known dose-dependent nature and frequency dependence of cisplatin-induced cochlear dysfunction, as well as to current ideas regarding harmonics detection and bone-conducted ultrasonic hearing. We also clarified that the dissociation observed in the present study places constraints on possible mechanisms without directly establishing one.

Comment: Minor comment 1. Some parts of the Results section repeat methodological details. These descriptions should be consolidated in the Methods section. Please also add references to the corresponding figures/tables at appropriate locations in the text.

Response: We agree and have streamlined the Results section. Details regarding the cisplatin regimen, timing of measurements, and justification for the regimen were consolidated in Materials and Methods. The Results section now focuses more directly on the findings, and figure references were checked and retained at the relevant locations.

Comment: Minor comment 2. In Figure 1C, the label “control” appears to refer to recordings obtained before cisplatin administration rather than a saline-treated control group. Please revise the labeling.

Response: We agree and have revised the wording in Figure 1C and its legend. The previous wording “control” was replaced with “before treatment,” and the corresponding paired traces are now described as recordings obtained before treatment and after cisplatin treatment.

Comment: Minor comment 3. Defining abbreviations such as conventional hearing range and bone-conducted ultrasonic stimulation would improve readability.

Response: We appreciate this suggestion. After careful consideration, we chose not to introduce additional abbreviations in the revised manuscript, because the number of core technical terms is limited and we felt that repeatedly spelling them out would maintain clarity for a broad readership. However, we revised the surrounding text to improve readability and reduce redundancy.

Response to Reviewer #2

Comment: General assessment: The study is well conceived and potentially impactful, but several methodological ambiguities and structural redundancies should be addressed.

Response: We thank the reviewer for the positive evaluation and constructive suggestions. We have revised the manuscript to clarify the calibration procedure, reduce overlap between Methods and Results, define the criteria used for wave III identification more explicitly, and discuss the relatively high baseline exclusion rate.

Comment: The method used to calibrate the bone-conducted ultrasonic stimulation is not sufficiently clear. Please explain how calibration was achieved using a hydrophone and clarify the rationale for using dB re 2 mPa in bone conduction.

Response: We agree and have expanded the calibration description in Materials and Methods. The revised text now explains that the hydrophone was used to calibrate the actuator output under the same driving conditions as those used in the experiment, thereby providing a standardized reference for stimulus intensity. We also clarified that the standard bone compression pressure of 2 mPa was used as a conventional reporting scale for bone-conducted stimulation rather than as a direct estimate of the mechanical load applied to the cochlea. In addition, we explicitly note that, although a minor airborne component could in principle contribute to stimulation, perforation of the tympanic membrane in our recordings minimized the contribution of air conduction.

Comment: There is noticeable redundancy between the Materials and Methods and Results sections, particularly regarding the descriptions of the experimental design and the cisplatin administration protocol.

Response: We agree and have revised the manuscript accordingly. Details of the experimental schedule, cisplatin dosing regimen, and the rationale for the regimen were consolidated in Materials and Methods, and the Results section was shortened so that it focuses more directly on the experimental findings.

Comment: Regarding the electrophysiological measurements, what specific criteria were used to determine the presence or absence of auditory brainstem response wave III? Please also provide information regarding intra-individual reproducibility and consistency.

Response: We have clarified this point in the Materials and Methods. The revised text now states that wave III was identified by visual inspection of the averaged auditory brainstem response waveforms as a repeatable positive-negative deflection occurring at the expected latency range under identical recording conditions, and that the same criterion was applied across animals and stimulus frequencies. Because thresholds were assigned from averaged responses recorded under identical conditions, this same standardized criterion was used consistently throughout the study.

Comment: The exclusion rate of mice at baseline due to pre-existing high-frequency hearing impairment appears relatively high. Please discuss possible causes, including whether environmental noise might have influenced baseline thresholds.

Response: We agree that this point warrants discussion. We have added a statement to the Discussion noting that the baseline exclusion rate due to pre-existing high-frequency hearing impairment was relatively high, that the cause of this pre-existing impairment was not determined in the present study, and that a predefined exclusion criterion was used to reduce this source of variability and to limit the analysis to animals meeting the baseline hearing requirement.

We again thank the Editor and the reviewers for their thoughtful and constructive comments. We hope that the revisions satisfactorily address all concerns and that the revised manuscript will now be suitable for publication in PLOS ONE.

Attachment Submitted filename: Response_to_Reviewers_PONE-D-26-20660.docx

# Peer review: Decision Letter 1

**DOI:** 10.1371/journal.pone.0353954.r003

**Authors:** Toru Miwa, Toru Miwa

1 Jul 2026

Bone-conducted ultrasonic auditory brainstem response thresholds in a mouse model of cisplatin-induced hearing loss

PONE-D-26-20660R1

Dear Dr. Nin,

We’re pleased to inform you that your manuscript has been judged scientifically suitable for publication and will be formally accepted for publication once it meets all outstanding technical requirements.

Within one week, you’ll receive an e-mail detailing the required amendments. When these have been addressed, you’ll receive a formal acceptance letter and your manuscript will be scheduled for publication.

An invoice will be generated when your article is formally accepted. Please note, if your institution has a publishing partnership with PLOS and your article meets the relevant criteria, all or part of your publication costs will be covered. Please make sure your user information is up-to-date by logging into Editorial Manager at Editorial Manager® and clicking the ‘Update My Information' link at the top of the page. For questions related to billing, please contact billing support.

If your institution or institutions have a press office, please notify them about your upcoming paper to help maximize its impact. If they’ll be preparing press materials, please inform our press team as soon as possible -- no later than 48 hours after receiving the formal acceptance. Your manuscript will remain under strict press embargo until 2 pm Eastern Time on the date of publication. For more information, please contact onepress@plos.org.

Kind regards,

Toru Miwa

Academic Editor

PLOS One

Additional Editor Comments (optional):

Reviewers' comments:

Reviewer's Responses to Questions

**Comments to the Author**

1. If the authors have adequately addressed your comments raised in a previous round of review and you feel that this manuscript is now acceptable for publication, you may indicate that here to bypass the “Comments to the Author” section, enter your conflict of interest statement in the “Confidential to Editor” section, and submit your "Accept" recommendation.

Reviewer #1: All comments have been addressed

Reviewer #2: All comments have been addressed

**********

2. Is the manuscript technically sound, and do the data support the conclusions?

The manuscript must describe a technically sound piece of scientific research with data that supports the conclusions. Experiments must have been conducted rigorously, with appropriate controls, replication, and sample sizes. The conclusions must be drawn appropriately based on the data presented.

Reviewer #1: Yes

Reviewer #2: (No Response)

**********

3. Has the statistical analysis been performed appropriately and rigorously?

Reviewer #1: Yes

Reviewer #2: (No Response)

**********

4. Have the authors made all data underlying the findings in their manuscript fully available?

The PLOS Data policy requires authors to make all data underlying the findings described in their manuscript fully available without restriction, with rare exception (please refer to the Data Availability Statement in the manuscript PDF file). The data should be provided as part of the manuscript or its supporting information, or deposited to a public repository. For example, in addition to summary statistics, the data points behind means, medians and variance measures should be available. If there are restrictions on publicly sharing data—e.g. participant privacy or use of data from a third party—those must be specified.

Reviewer #1: Yes

Reviewer #2: (No Response)

**********

5. Is the manuscript presented in an intelligible fashion and written in standard English?

PLOS ONE does not copyedit accepted manuscripts, so the language in submitted articles must be clear, correct, and unambiguous. Any typographical or grammatical errors should be corrected at revision, so please note any specific errors here.

Reviewer #1: Yes

Reviewer #2: (No Response)

**********

6. Review Comments to the Author

Please use the space provided to explain your answers to the questions above. You may also include additional comments for the author, including concerns about dual publication, research ethics, or publication ethics. (Please upload your review as an attachment if it exceeds 20,000 characters)

Reviewer #1: I thank the authors for their careful and constructive responses to the review comments. The manuscript has been substantially improved. In particular, the Introduction and Discussion now more clearly define the study objective and appropriately position the work as a functional and phenomenological investigation rather than a mechanistic study. The discussion has also been strengthened by relating the findings to the known pathophysiology of cisplatin-induced cochlear damage and current hypotheses regarding bone-conducted ultrasonic hearing. The remaining revisions have been appropriately addressed, and I have no further major concerns. I believe the manuscript is suitable for publication in its current form.

Reviewer #2: (No Response)

**********

7. PLOS authors have the option to publish the peer review history of their article (what does this mean?). If published, this will include your full peer review and any attached files.

If you choose “no”, your identity will remain anonymous but your review may still be made public.

**Do you want your identity to be public for this peer review?**  For information about this choice, including consent withdrawal, please see our Privacy Policy.

Reviewer #1: No

Reviewer #2: **Yes:**  Takuji KOIKE

**********

# Editor report: Acceptance letter

**DOI:** 10.1371/journal.pone.0353954.r004

**Authors:** Toru Miwa, Toru Miwa

PONE-D-26-20660R1

PLOS One

Dear Dr. Nin,

I'm pleased to inform you that your manuscript has been deemed suitable for publication in PLOS One. Congratulations! Your manuscript is now being handed over to our production team.

At this stage, our production department will prepare your paper for publication. This includes ensuring the following:

* All references, tables, and figures are properly cited

* All relevant supporting information is included in the manuscript submission,

* There are no issues that prevent the paper from being properly typeset

You will receive further instructions from the production team, including instructions on how to review your proof when it is ready. Please keep in mind that we are working through a large volume of accepted articles, so please give us a few days to review your paper and let you know the next and final steps.

Lastly, if your institution or institutions have a press office, please let them know about your upcoming paper now to help maximize its impact. If they'll be preparing press materials, please inform our press team within the next 48 hours. Your manuscript will remain under strict press embargo until 2 pm Eastern Time on the date of publication. For more information, please contact onepress@plos.org.

You will receive an invoice from PLOS for your publication fee after your manuscript has reached the completed accept phase. If you receive an email requesting payment before acceptance or for any other service, this may be a phishing scheme. Learn how to identify phishing emails and protect your accounts at https://explore.plos.org/phishing.

If we can help with anything else, please email us at customercare@plos.org.

Thank you for submitting your work to PLOS ONE and supporting open access.

Kind regards,

PLOS ONE Editorial Office Staff

on behalf of

Dr. Toru Miwa

Academic Editor

PLOS One
